# Dual Wavelength Photoplethysmography Framework for Heart Rate Calculation

**DOI:** 10.3390/s22249955

**Published:** 2022-12-17

**Authors:** Ludvik Alkhoury, JiWon Choi, Vishnu D. Chandran, Gabriela B. De Carvalho, Saikat Pal, Moshe Kam

**Affiliations:** 1 Department of Electrical and Computer Engineering, Newark College of Engineering, New Jersey Institute of Technology, Newark, NJ 07102, USA; 2 Department of Biomedical Engineering, Newark College of Engineering, New Jersey Institute of Technology, Newark, NJ 07102, USA

**Keywords:** photoplethysmography (PPG), dual-wavelength PPG, green and infrared PPG, motion artifacts reduction, heart rate calculation

## Abstract

The quality of heart rate (HR) measurements extracted from human photoplethysmography (PPG) signals are known to deteriorate under appreciable human motion. Auxiliary signals, such as accelerometer readings, are usually employed to detect and suppress motion artifacts. A 2019 study by Yifan Zhang and his coinvestigatorsused the noise components extracted from an infrared PPG signal to denoise a green PPG signal from which HR was extracted. Until now, this approach was only tested on “micro-motion” such as finger tapping. In this study, we extend this technique to allow accurate calculation of HR under high-intensity full-body repetitive “macro-motion”. Our Dual Wavelength (DWL) framework was tested on PPG data collected from 14 human participants while running on a treadmill. The DWL method showed the following attributes: (1) it performed well under high-intensity full-body repetitive “macro-motion”, exhibiting high accuracy in the presence of motion artifacts (as compared to the leading accelerometer-dependent HR calculation techniques TROIKA and JOSS); (2) it used only PPG signals; auxiliary signals such as accelerometer signals were not needed; and (3) it was computationally efficient, hence implementable in wearable devices. DWL yielded a Mean Absolute Error (MAE) of 1.22|0.57 BPM, Mean Absolute Error Percentage (MAEP) of 0.95|0.38%, and performance index (PI) (which is the frequency, in percent, of obtaining an HR estimate that is within ±5 BPM of the HR ground truth) of 95.88|4.9%. Moreover, DWL yielded a short computation period of 3.0|0.3 s to process a 360-second-long run.

## 1. Introduction

Multi-diagnostic wearable devices are of ongoing interest due to their ability to store and transmit information about the wearer inexpensively and efficiently. Many wearable sensors employ photoplethysmography (PPG), a low-cost optical technique used to detect blood volume changes in the microvascular bed of tissues [1]. This technique enables noninvasive detection of the cardiovascular pulse wave generated by the elastic nature of the peripheral vascular arteries excited by the quasi-periodic contractions of the heart [2,3,4]. PPG signals are used in pulse oximeters—devices that measure the light absorbed by functional hemoglobin (oxygenated and deoxygenated hemoglobin) and produce vital signs such as heart rate (HR) or peripheral capillary oxygen saturation ($SpO_{2}$) (an estimate of the arterial oxygen saturation ($SaO_{2}$) [5]). In order to obtain a PPG signal, light is typically shone through the skin and its reflection is captured by a photo-detector. In this study, we serially collect the reflection of light at two different wavelengths, namely, green and infrared (IR).

In the presence of substantial human motion, the quality of the measured PPG signal deteriorates [6]. Much effort has been exerted to suppress motion artifacts in order to extract high-quality vital signs from noise-contaminated PPG signals [3,7,8,9,10]. This study contributes to this effort.

There are two main sources of motion artifacts that could contaminate a PPG signal collected from a human in motion [9]. The first source of noise is the sensor displacement relative to its original point of contact with the skin. This displacement could alter the path of light, and hence modify the signal collected by the photo-detector [11]. The second source of noise is skin and tissue deformations caused by the sensor’s movement.

Zhang et al. [9] proposed an HR calculation method that uses a dual-wavelength sensor that comprises an IR and a green PPG signal. The IR PPG signal was employed to develop a noise source that was used to denoise the green PPG signal from which an HR level was extracted.

The HR calculation algorithm presented in [9] was tested on “micromotion artifacts” such as “finger tapping” and “fist opening and closing”. In the current study, we examined the applicability of a related approach for more substantial movements and dynamic scenarios. Motivated by the sensor architecture proposed in [9], we expanded the HR calculation technique to high-intensity full-body repetitive “macro-motion” exercise data. The resulting Dual Wavelength (DWL) method collects green and IR PPG data from a dual-wavelength wrist unit and processes them to estimate the participant’s heart rate. The performance of DWL was documented in an extensive motion experiment involving fourteen (14) human participants. There were three separate experiments. In the first (SNR experiment), we used all fourteen (14) participants. In the second experiment (wrist-based heart rate calculation), we used eleven (11) participants due to sensor failure on three of the participants. In the third experiment (palm-based heart rate calculation), we used twelve (12) participants due to sensor failure on two of the participants.

**Figure 1:**
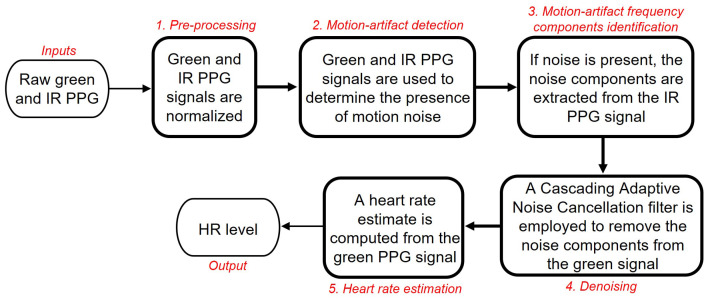
Summary of the DWL method.

Figure 1 shows the essentials of the DWL method. It consists of five (5) stages; *1. Pre-processing*, *2. Motion-artifact detection*, *3. Motion-artifact frequency components identification*, *4. Denoising*, and *5. Heart rate estimation*. The inputs to the DWL system are green and IR PPG channels measured from a wrist-unit constructed for this study (see Section 2.1). The output is an HR level. First, the green and IR PPG signals are normalized by dividing the signal’s AC component by its DC component. We then check whether significant motion noise is present in the PPG signals (Section 2.3.2). If the signals appear noise-free, the normalized green PPG signal is directly used to calculate an HR value. If the signals appear noise contaminated, we then extract the noise components from the IR PPG signal. These noise components are removed from the noisy green PPG signal. We employ a Cascading Adaptive Noise Cancellation (C-ANC) architecture that uses a QR-decomposition-based least-squares lattice (QRD-LSL) algorithm [12] to denoise the green PPG signal before it is used for HR calculation. A separate decision mechanism validates the HR estimate, and corrects it when noise levels are excessively high to produce a meaningful estimate.

The rest of this paper is organized as follows. In Section 2, we present the materials and methods we employ in this study. Section 2.1 describes the experimental settings along with the sensors’ suite. In Section 2.2, we use experimental data to present the rationale for choosing the IR PPG signal as noise reference signal. Section 2.3 introduces the DWL framework; a method for (1) denoising the green PPG using the noise components extracted from an IR PPG signal, and (2) computing HR levels. Lastly, in Section 2.4, we review alternative HR calculation methods that use auxiliary sensors as a noise source, namely accelerometers. These methods are TROIKA [7] and JOSS [8]. Section 3 presents the results of the DWL framework. In Section 3.1, we define the performance metrics used to compare the performance of the DWL method to that of our implementations of TROIKA and JOSS. In Section 3.2, we compare the actual performance of the DWL method to that of our implementations of TROIKA and JOSS. The comparison is made with respect to (1) the heart rate ground truth computed from an electrocardiography (ECG) signal, and (2) the heart rate levels obtained using TROIKA and JOSS. Section 3.3 validates the DWL framework by testing its performance on experimental data collected from the palms (instead of wrists) of the same participants during a second run (validation run). In Section 4, we conclude that the DWL method provides several desirable features, including the following: (1) the DWL framework uses only PPG signals; auxiliary signals (such as accelerometers used by TROIKA and JOSS) are not needed and (2) the DWL framework appears to exhibit high accuracy and lower computational burden in the presence of motion artifacts as compared to TROIKA and JOSS.

## 2. Materials and Methods

In this section, the materials and methods employed in this work are presented. In Section 2.1, we present the sensors used for data collection. We also describe the exercise protocol followed during data collection. In our framework, noise components are extracted from an IR PPG signal. In Section 2.2, we show using experimental data, the rationale behind the choice of IR PPG signal as a reference noise source. In Section 2.3, the DWL framework is introduced and described in detail (along with its five (5) stages, namely, *pre-processing*, *Motion-artifact detection*, *Motion-artifact frequency components identification*, *Denoising*, and *Heart rate estimation*). We compare the performance of the DWL method to alternative HR calculation methods that use auxiliary sensors as a noise source, namely accelerometers. These methods are TROIKA [7] and JOSS [8]. In Section 2.4, we present the framework of these two alternative HR calculation methods.

### 2.1. Experimental Protocol and Sensors Suite

We conducted a high-intensity full-body exercise experiment where we collected PPG, electrocardiography (ECG), and tri-axial accelerometer data. Accelerometers measured accelerations in three orthogonal directions X, Y, and Z, simultaneously [13]. Readings were obtained from fourteen (14) human participants while they were standing or running on a split-belt instrumented treadmill (Bertec Corp., Columbus, OH) [14]. First, a multi-wavelength wrist oximeter unit was strapped around the participant’s wrist. The wrist unit encloses two green LEDs (of wavelength $\lambda_{G}=520$ nm) and two IR LEDs (of wavelength $\lambda_{IR}=940$ nm), as well as a photo-detector. Additionally, a tri-axial accelerometer sensor was placed on the participant’s arm (right above the PPG wrist-unit) and secured in place using athletic tape. Lastly, an ECG sensor was mounted onto the participant’s chest using adhesive electrodes. Athletic tape was wrapped around each participant’s chest to ensure the sensor’s stability and good skin contact. Table 1 shows all the instruments and sensors used in the experiment. Both ECG and accelerometer data were recorded using the Delsys EMGworks Software. Multi-wavelength PPG wrist-unit data were recorded using an Arduino UNO. All signals were sampled at 100 Hz. Raw data were processed using MATLAB 2022b (Mathworks, Natick, MA) [15]. All raw data are available through the Github repository in [16].

The ECG signal was used to calculate the HR “ground truth” values. We manually labeled the *R* peaks for all ECG signals. The HR ground truth at time step *l*, $HR_{GT}\left(l\right)$, is obtained using the relationship

(1) $$HR_{GT}\left(l\right)=\frac{1}{\delta^{R-R}\left(l\right)},$$

where $\delta^{R-R}\left(l\right)$ is the average time difference between each two consecutive *R* peaks present within the 8-second-long window, at time step *l*.

The experimental protocol we followed during data collection was conducted in accordance with the Declaration of Helsinki, and approved by the Institutional Review Board of the New Jersey Institute of Technology (protocol code 2108010504; approved on 14 September 2021). All participants were physically fit, healthy, and athletic volunteers. Each participant was asked to run on a treadmill following an exercise profile that comprises six (6) stages. At each stage, the treadmill speed—hence the exercise intensity—was varied as follows:

- Stage 1: The participant stood steady on the treadmill for 1 min (here, the treadmill’s speed was 0 km/h). During this stage, clean physiological signals were collected.
- Stage 2: The participant ran at a speed of 6 km/h (about 3.7 mph) for 1 min (here, the treadmill’s speed was 6 km/h).
- Stage 3: If the participant was comfortable, the treadmill’s speed was increased gradually to 12 km/h (about 7.5 mph), for 1 min. At any time, if the participant was not comfortable, the treadmill’s speed was reduced to the participant’s comfort zone.
- Stage 4 (same as stage 2): The participant ran at a speed of 6 km/h (about 3.7 mph) for 1 min (here, the treadmill’s speed was 6 km/h).
- Stage 5 (same as stage 3): If the participant was comfortable, the treadmill’s speed was increased gradually to 12 km/h (about 7.5 mph), for 1 min. At any time, if the participant was not comfortable, the treadmill’s speed was reduced to the participant’s comfort zone.
- Stage 6: The participant stood steady on the treadmill for a duration of 1 min (here, the treadmill’s speed was 0 km/h).

### 2.2. Infrared PPG Signal as Noise Reference Signal

According to [9], IR PPG signals are more affected by motion artifacts than green PPG signals. To verify this behavior in our experiment, we calculated the signal-to-noise (SNR) ratios for both the green and IR PPG signals. The SNR is defined as

(2) $$SNR\left(in\;dB\right)=10\;log_{10}\left(\frac{P_{desired\;signal}}{P_{noise}}\right),$$

where $P_{desired\;signal}$ and $P_{noise}$ are the power of the participant’s heart rate component and motion artifact components, respectively. In order to calculate an SNR value, the desired and noise signal components should be identified and separated. At this stage, we used the participant’s HR ground truth (obtained from an ECG signal, collected simultaneously with the PPG signals) using Equation (1), in order to determine the desired signal component.

The desired signal and noise components were obtained, respectively, from the green and IR PPG signals. First, the green and IR signals were normalized by dividing their AC component by their DC component. The desired signal component (the component that contains heart rate information) of the normalized PPG signal was obtained by applying two bandpass filters centered at the participant’s HR frequency (fundamental frequency) and its second harmonic [9]. During this step the participant’s HR was obtained from the ECG signal. The noise component was obtained by subtracting the desired signal component from the normalized signal.

**Figure 2:**
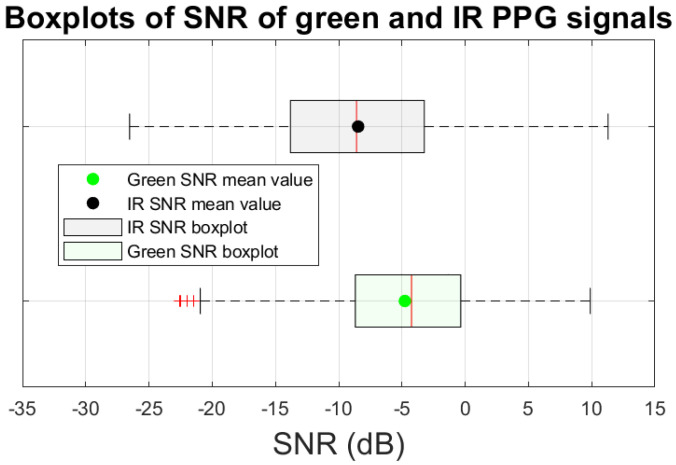
SNR values of IR and green PPG signals, respectively, calculated from all fourteen (14) participants. The dots represent the mean value of SNR. The red bars represent the median value of SNR. The red ‘+’ signs represent outliers.

We calculated the SNR values of the green and IR PPG signals for all fourteen (14) participants in the following manner. Every 2 s, the preceding 8-second-long PPG segment was used to obtain an SNR value. In total, each participant had between 175 and 177 SNR values for each PPG signal (green and IR signals). The first and last minute of the collected PPG data were omitted since these data segments were noise-free. SNR values for all participants were grouped together and their distribution is presented in Figure 2 as boxplots [22].

In our experiment, the SNR mean value of the IR PPG, $\mu_{SNR}^{IR}$ = −8.5 dB (black dot in Figure 2), was less than the SNR mean value of the green PPG signal, $\mu_{SNR}^{G}$ = −4.8 dB (green dot in Figure 2). These results are statistically significant for a level of significance α=0.01. This difference supports the choice of IR PPG as a noise reference signal using experimental data.

### 2.3. DWL Framework

The proposed DWL framework consists of the following stages (Figure 1), *A. Pre-processing*, *B. Motion-artifact Detection*, *C. Motion-artifact Frequency Components Identification*, *D. Denoising*, and *E. Heart Rate Estimation*. The inputs to the system are raw green and IR PPG signals measured using the dual-wavelength PPG wrist-unit sensor (described in Section 2.1). The output is an HR estimate, $\hat{HR}\left(l\right)$ at time step *l* (the initial time step is l=1). We refer to the average of the latest *Z* estimates of the heart rate as ${\hat{HR}}^{\left(Z\right)}\left(l\right)$, namely

(3) $${\hat{HR}}^{\left(Z\right)}\left(l\right)=\frac{1}{Q}\sum_{q=0}^{Q-1}\hat{HR}\left(l-q\right)\;|\;Q=min\left\{Z,l\right\}.$$

**Figure 3:**
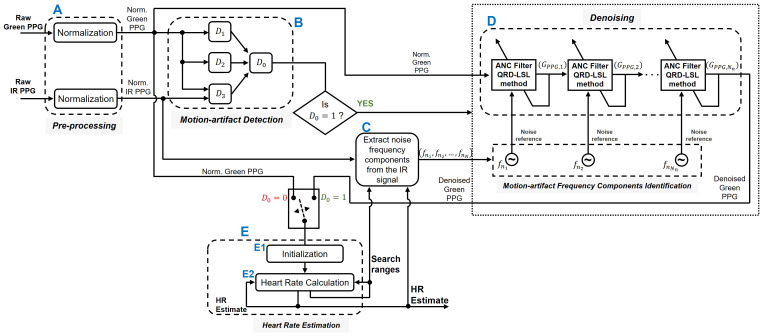
Block diagram of the DWL method. The inputs for calculating HR are raw green and IR PPG signals. The output is an estimate of the participant’s HR. Block A corresponds to the pre-processing stage of Section 2.3.1. Block B represents the motion-artifact detection stage of Section 2.3.2, which we employed to determine whether the PPG signals are contaminated with appreciable level of noise. Block C identifies the motion-artifact frequency components as described in Section 2.3.3. During this stage, noise frequency components are extracted from the IR PPG signal. Block D is the denoising stage of Section 2.3.4, during which noise components are removed from the green PPG signal. Block E corresponds to the heart rate estimation stage of Section 2.3.5, which we used to extract an HR estimate from the green PPG signal. Block E1 is the HR “Initialization” stage, during which the initial HR value is computed. Block E2 illustrates the motion-resistant HR calculation mechanism.

Figure 3 is a block diagram of the DWL method. The system produced a new estimate of HR at every time step ($\hat{HR}\left(l\right)$ at time step *l*). The time between two subsequent windows in our study was 2 s. In addition, the system produces three *search ranges*. They are; the “narrow search range”, $\Delta_{n}\left(l+1\right)$; the “medium search range”, $\Delta_{m}\left(l+1\right)$; and the “wide search range”, $\Delta_{w}\left(l+1\right)$. Ranges $\Delta_{m}\left(l+1\right)$ and $\Delta_{n}\left(l+1\right)$, which are used in the motion-artifact frequency components identification process of Section 2.3.3, are centered at $\hat{HR}\left(l\right)$. The range $\Delta_{w}\left(l+1\right)$, which is used in the heart rate estimation process of Section 2.3.5, is centered at ${\hat{HR}}^{\left(6\right)}\left(l\right)$, the average of the 6 previous heart rate estimates. The ranges satisfy $\Delta_{n}\left(l+1\right)<\Delta_{m}\left(l+1\right)<\Delta_{w}\left(l+1\right)$. Moreover, $\Delta_{n}\left(l+1\right)=\frac{\Delta_{m}\left(l+1\right)}{2}$ (for details on how we calculated $\Delta_{w}\left(l+1\right)$ and $\Delta_{m}\left(l+1\right)$, see Equations (A2) and (A4) in Appendix A, respectively). Lastly, we calculate a short-term 3-point-average heart rate, ${\hat{HR}}^{\left(3\right)}\left(l\right)$, that we provide to the users and employ in Section 3.2 for assessing the performance of DWL.

**Figure 4:**
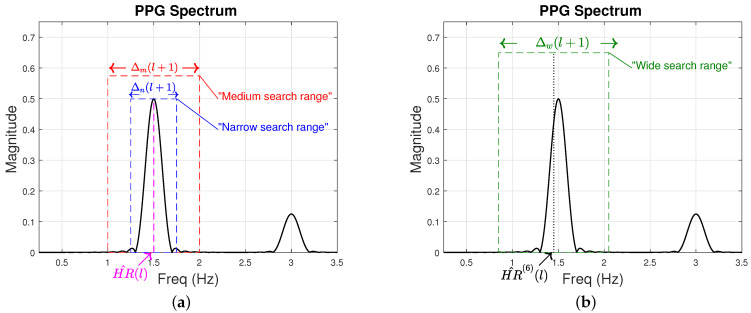
Illustration of the frequency spectrum of typical IR PPG signal. (**a**) The narrow search range, $\Delta_{n}\left(l+1\right)$ and the medium search range, $\Delta_{m}\left(l+1\right)$ are illustrated as a blue and a red dashed rectangle, respectively. Both ranges are centered around $\hat{HR}\left(l\right)=1.5$ Hz which is shown as a magenta dashed line. These ranges are used for noise frequency component search. (**b**) The wide search range, $\Delta_{w}\left(l+1\right)$ is illustrated as a green dashed rectangle. The wide search range is centered at ${\hat{HR}}^{\left(6\right)}\left(l\right)$ (black dotted line) and is used to search, at time step l+1, for $\hat{HR}\left(l+1\right)$.

Figure 4 is an illustration of a typical IR PPG spectrum. The magenta dashed line in Figure 4a is the heart rate estimated at time step *l*, $\hat{HR}\left(l\right)$. The black dotted line in Figure 4b is the average of the 6 previous heart rate estimates at time step *l*, ${\hat{HR}}^{\left(6\right)}\left(l\right)$. In this example, $\hat{HR}\left(l\right)$ is 1.5 Hz and ${\hat{HR}}^{\left(6\right)}\left(l\right)$ is 1.45 Hz. Additionally, we present in Figure 4 the “wide search range”, $\Delta_{w}\left(l+1\right)$, as a green dashed rectangle, the “medium search range”, $\Delta_{m}\left(l+1\right)$, as a red dashed rectangle, and the “narrow search range”, $\Delta_{n}\left(l+1\right)$, as a blue dashed rectangle.

#### 2.3.1. Pre-Processing

First, both green and IR PPG signals are normalized (block A of Figure 3). Normalization is done by dividing the signal’s AC component by its DC component [23]. The AC component is obtained by passing the raw PPG signal through a Chebyshev Type II bandpass filter of order 5 and bandpass frequency range from 0.5 to 10 Hz. The DC component is obtained by passing the raw signal through a Chebyshev Type II low-pass filter of order 5 and passband frequency of 0.5 Hz.

#### 2.3.2. Motion-Artifact Detection

Motion artifact detection is used to determine whether the PPG signals are contaminated by motion noise (if they are not, we can bypass unnecessary noise suppression operations). The PPG signals go through the following three (3) local detectors to determine if appreciable levels of noise motion are present (block B of Figure 3):

Local Detector 1 ($D_{1}$)—Number of Peaks: The number of dominant peaks (whose magnitude exceeds 30% of the maximum peak for this example) in the frequency spectrum of the green PPG signal, denoted $N_{p}$, is calculated. If $N_{p}$ exceeds two (2), $D_{1}$ indicates that the signal is contaminated with motion noise. If $N_{p}$ is 1 or 2, then we conclude that no appreciable motion noise is present, since the frequency of the heart rate and sometimes its second harmonic component are typically observed in the spectrum of a clean PPG signal.

Local Detector 2 ($D_{2}$)—Power of Green Signal: The power of the green PPG signal calculated at the beginning of the experiment (when the participant is at rest) is considered the reference power, denoted $P^{ref}$. At each time step *l*, the power of the green PPG, $P^{G}\left(l\right)$, is calculated and compared to the reference power $P^{ref}$. If $P^{G}\left(l\right)$ is more than $\left(1+\kappa \right)P^{ref}$, $D_{2}$ indicates that the green PPG signal is contaminated with motion noise. The amplitude of the PPG signal might change over time [24]. Therefore, the reference power $P^{ref}$ is updated whenever no motion is detected in the system for five (5) consecutive time steps (global detector $D_{0}$ return ‘1’). In this case, the updated value of $P^{ref}$ is set to the power of the green PPG signal calculated at the current time step, *l*. In this study, we used κ=0.2.

Local Detector 3 ($D_{3}$)—Pearson Correlation between Green and IR PPG Signals: The correlation between the green and IR PPG signals is also used to assess noise contamination in the green signal. If the correlation between the green and IR PPG signals, $\rho_{green,\;IR}$, is below a certain threshold (we used 0.8), then $D_{3}$ will decide that the green PPG signal is contaminated with motion noise.

Global Detector—Noise Detector: The decisions of the three local detectors are fed into a global detector that will decide whether the signal is noise contaminated. The global detector is shown as:

(4) $$D_{0}=D_{1}\vee D_{2}\vee D_{3}=\left\{\begin{array}{cc} 1\;(noise\;is\;present) & ,\mathrm{if}\;D_{1}\vee D_{2}\vee D_{3}=1\\ 0\;(no\;noise) & ,\mathrm{if}\;D_{1}\vee D_{2}\vee D_{3}=0 \end{array}\right.$$

where “∨” represents the OR logic operator.

#### 2.3.3. Motion-Artifact Frequency Components Identification

If motion artifacts are detected in the normalized green PPG signal, we use the normalized IR signal to build the motion noise component set $N_{noise}$ (block C of Figure 3). $N_{noise}$ can be written as $N_{noise}=\left\{f_{n_{i}}|1\leq i\leq N_{n}\right\}$ where $f_{n_{i}}$ is the $i^{th}$ discrete noise frequency component and $N_{n}$ is the number of elements in the set $N_{noise}$. The set $N_{noise}$, which contains all the noise frequency components that we aim to remove from the normalized green PPG signal, is obtained using the following five (5) steps in sequence. The first three steps capture noise with relatively high intensity, usually harmonically related frequency pairs that contaminate the PPG signals. The last two steps compare the IR and green signal spectra to discover additional noise components of reduced-intensity presence in the IR spectrum.

**Figure 5:**
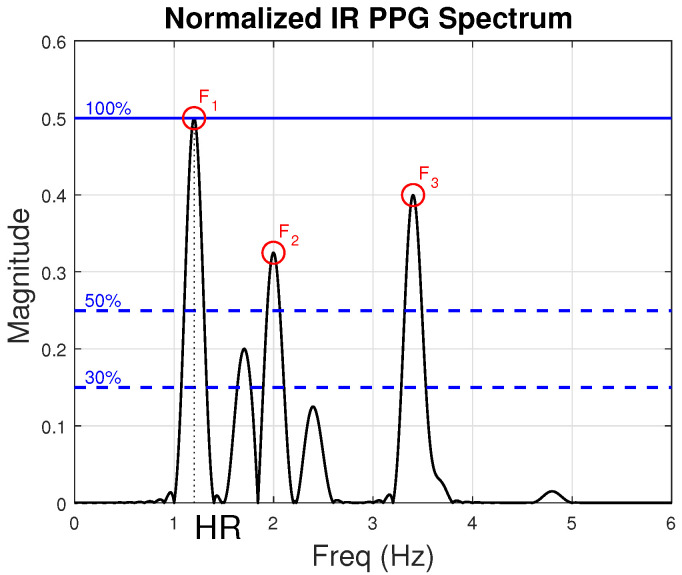
Illustration of the frequency spectrum of a typical IR PPG signal. The red circles correspond to the dominant peaks, denoted $F_{1}$, $F_{2}$, and $F_{3}$ (extracted in step 1 of Section 2.3.3). The highest peak, $F_{1}$, corresponds to the participant’s HR.

Step 1—Identification of dominant frequency components. First, we capture the dominant frequency components in the spectrum of the normalized IR PPG signal. Those are the frequencies (between 0.5 and 4 Hz) whose magnitude exceeds 50% of the highest peak in the IR PPG spectrum. Figure 5, which is an image that was created for illustration purposes, depicts how we capture dominant peaks from a typical IR signal. In this scenario, the highest peak (which actually corresponds to the participant’s HR) is $F_{1}$. Two other dominant peaks are shown as red circles ($F_{2}$ and $F_{3}$). Typically, the peaks captured in step 1 include the frequency of the participant’s HR, as well as the frequencies of dominant noise components. We add all of them ($F_{1}$, $F_{2}$, and $F_{3}$ in our example) to $N_{noise}$ with the understanding that one of them may correspond to the participant’s HR and may therefore need to be removed from $N_{noise}$ later.

**Figure 6:**
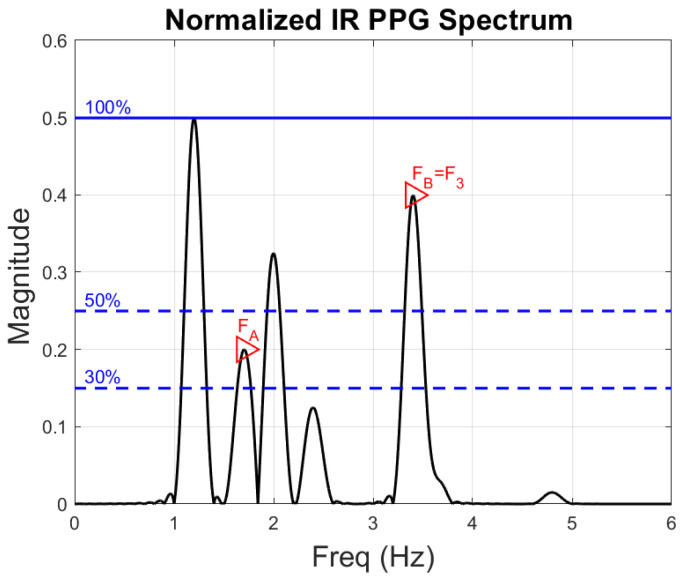
Illustration of the frequency spectrum of a typical IR PPG signal. The red triangles correspond to the pair of frequencies, $F_{A}$ and $F_{B}$, that has a harmonic relationship. Frequency $F_{B}$ is the same as frequency $F_{3}$ from Figure 5.

Step 2—Identification of harmonic frequency components. Noise components created by repetitive motion (e.g., when the participant is walking or running) typically occur in harmonically related pairs [25]. It is possible, however, that the PPG signal contains pairs of harmonically related noise components whose magnitude is smaller than the 50% threshold used in step 1 to identify dominant frequencies. Step 2 is used to capture pairs of fundamental frequencies and their second harmonics present in the spectrum of the normalized IR PPG signal. Here, we look at all peaks whose magnitudes are above 30% of the highest peak in the IR PPG spectrum. For each such peak, we search for a harmonic at double its frequency. If a pair of harmonically related frequencies is thus discovered, its component(s) that were not flagged in step 1 are added to the noise frequency set $N_{noise}$. Again, $N_{noise}$ may still contain at this stage a component that corresponds to the participant’s true HR. Figure 6 uses the same spectrum shown in Figure 5 to illustrate how a pair of harmonically related components ($F_{A}$, $F_{B}=F_{3}$) was discovered. Of this pair, $F_{B}$ was known to us already from step 1 (it is the same as $F_{3}$ in Figure 5), and $F_{A}$, discovered by step 2, is added to $N_{noise}$. So now, $N_{noise}=\left\{F_{1},F_{2},F_{3},F_{A}\right\}$.

Step 3—Removal of the heart rate from noise set. As mentioned in our setting in Section 2.3, our system creates a new estimate of the heart rate, $\hat{HR}\left(l\right)$ at every time step *l*. A new time step starts every 2 s when *l* is incremented by 1. Moreover, in step l+1 we calculate $\Delta_{w}\left(l+1\right)$ (the “wide search range”) which is where we search for $\hat{HR}\left(l+1\right)$.

Next, frequency components in $N_{noise}$ which we captured during steps 1 and 2, and are close to the heart rate estimated at time step *l* ($\hat{HR}\left(l\right)$) are removed from $N_{noise}$, as we suspect they do not represent noise but rather represent the participant’s HR. To be precise, at time step l+1, we remove from $N_{noise}$ all the noise components in the “medium search range” $\Delta_{m}\left(l+1\right)$.

**Figure 7:**
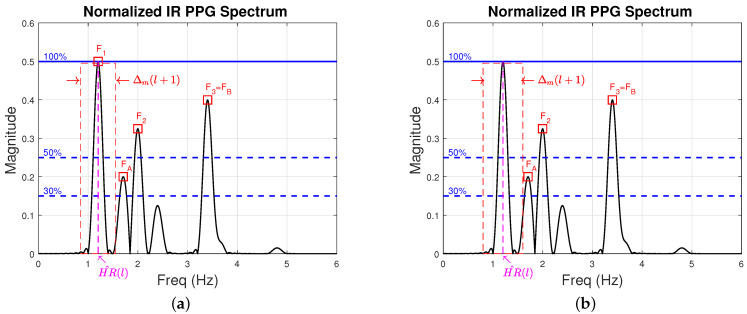
Frequency spectrum of a typical IR PPG signal. $\hat{HR}\left(l\right)$ is the heart-rate estimate at time step *l*. $\Delta_{m}\left(l+1\right)$ is the “medium search range” represented by a red dashed rectangle. The frequency components we obtained from step 1 and 2, namely, $F_{1}$, $F_{A}$, $F_{2}$, and $F_{3}=F_{B}$, are represented by red squares. In (**a**) frequency $F_{1}$ falls within $\Delta_{m}\left(l+1\right)$. In (**b**) we discard the frequency $F_{1}$ since it falls within $\Delta_{m}\left(l+1\right)$ and leave the rest in $N_{noise}$ ($F_{A}$, $F_{2}$, and $F_{3}=F_{B}$).

Figure 7 continues the examples of Figure 5 and Figure 6 to illustrate step 3. In Figure 7a,b, we show the estimate of the participant’s HR at time step *l*, denoted $\hat{HR}\left(l\right)$. We also show $\Delta_{m}\left(l+1\right)$, the “medium search range”, $\left[\hat{HR}\left(l\right)-\Delta_{m}\left(l+1\right)/2,\hat{HR}\left(l\right)+\Delta_{m}\left(l+1\right)/2\right]$, from which we remove dominant frequencies deposited earlier into $N_{noise}$. The red squares in Figure 7a represent the frequency components that we obtained from steps 1 and 2 all of which are currently in $N_{noise}=\left\{F_{1},F_{2},F_{3},F_{A}\right\}$. We now discard the frequency around 1.2 Hz (labeled $F_{1}$) since it falls in $\Delta_{m}\left(l+1\right)$, the “medium search range” (region represented by a red dashed rectangle in Figure 7). Figure 7b shows (in red squares) the noise frequency components that are left in the noise set $N_{noise}=\left\{F_{2},F_{3},F_{A}\right\}$. $N_{noise}$ no longer contains the participant’s HR.

The next two steps seek additional noise components, often attributed to repetitive movements by the participant, through comparison of the IR and green spectra.

Step 4: Step 4 focuses on instances where the noise set $N_{noise}$, after step 3, has only one noise component, $f_{n_{1}}$. In this case, we look at the green spectrum. If we find a component at half $f_{n_{1}}$ ($f_{n_{1}}/2$) or twice $f_{n_{1}}$ ($2\times f_{n_{1}}$) in the green spectrum, we add this component to $N_{noise}$. The only exception is if the component we seek to add falls into the *narrow search range*, $\Delta_{n}\left(l+1\right)$, around $\hat{HR}\left(l\right)$, $\left[\hat{HR}\left(l\right)-\Delta_{n}\left(l+1\right)/2,\hat{HR}\left(l\right)+\Delta_{n}\left(l+1\right)/2\right]$; in this case, we refrain from adding it to set $N_{noise}$.

Step 5: This step addresses spectra that are dominated by vigorous limb swinging by the participant, which may cause displacement of the sensor. In this scenario, the green PPG signal is typically dominated by two high intensity harmonically related noise frequencies which may dwarf the component at the heart rate frequency. If these frequency components are not already placed in $N_{noise}$ after steps 1–3, they are added to $N_{noise}$ at this step. This step is automatically triggered when all the following conditions are met, namely; (a) the IR spectrum contains only one significant frequency component that dominates the spectrum; (b) the green spectrum contains only one pair of significant harmonically related frequencies; and (c) the dominant frequency component present in the IR spectrum matches one of the harmonically related frequencies discovered in the green spectrum.

**Figure 8:**
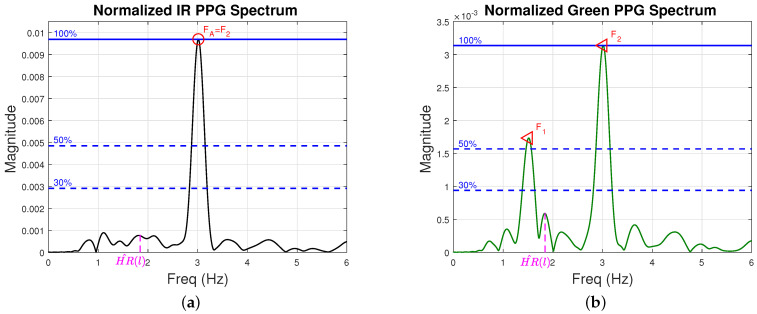
IR and green spectra from participant 10 around 136 s. We show in magenta the heart rate estimate at time step *l*, $\hat{HR}\left(l\right)$. (**a**) Frequency spectrum of participant 10’s IR PPG signal. The red circle labeled $F_{A}=F_{2}$ represent the dominant noise frequency. (**b**) Frequency spectrum of participant 10’s green PPG signal around time 136 s. The two red triangles labeled $F_{1}$ and $F_{2}$ represent high-intensity harmonically related frequencies. Note that the frequency $F_{A}$ from (**a**) is the same of the frequency $F_{2}$ from subplot (**b**). Both $F_{1}$ and $F_{A}=F_{2}$ are put into $N_{noise}$.

Figure 8 is a real-life example that illustrates this scenario (signals were collected from participant 10 in our experiment, around time 136 s). We show the spectrum of participant 10’s IR signal in Figure 8a and green signal in Figure 8b. We show in magenta the heart rate estimate at time step *l*, $\hat{HR}\left(l\right)$. The green signal captures the high-intensity harmonically related frequency pairs $F_{1}$ and $F_{2}$ of Figure 8b. The IR spectrum (Figure 8a) is dominated by the frequency $F_{A}$ that is equal to frequency $F_{2}$ from the green spectrum, but does not capture a noise component at $F_{1}$. Here, frequencies $F_{1}$ and $F_{A}=F_{2}$ are put into $N_{noise}$.

At the end of this stage, the set $N_{noise}$ will contain $N_{n}$ elements that correspond to the noise frequencies we wish to remove from the normalized green PPG signal.

#### 2.3.4. Denoising

Adaptive Noise Cancellation (ANC) filters are often employed to eliminate in-band motion artifacts [26,27]. In-band noise in our case occurs when the spectra of motion artifacts overlap significantly with that of the PPG signal [28]. An ANC filter for our environment would use as inputs (1) a noise contaminated signal, and (2) a noise reference signal. The ANC filter seeks to eliminate the noise components (measured by the reference signal) from the input noise contaminated signal and provide a noise-free version of the input signal.

**Figure 9:**
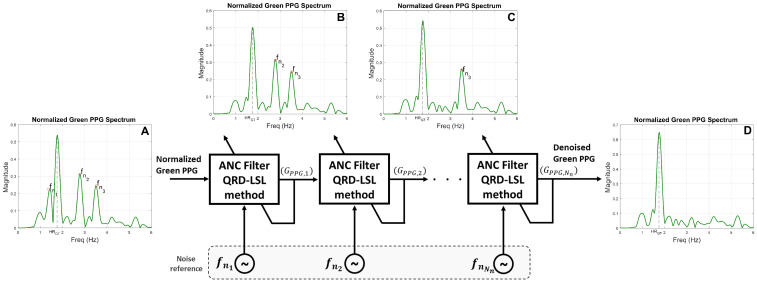
Cascading Adaptive Noise Canceler (C-ANC) block diagram. Spectrum A is of the noise-contaminated green PPG signal which is fed to the C-ANC. Spectrum B represents the green PPG signal’s frequency spectrum after removal of the first noise frequency component, $f_{n_{1}}$. Spectrum C is obtained after removing a second noise frequency component, $f_{n_{2}}$. At this stage, $f_{n_{1}}$ and $f_{n_{2}}$ are removed from the input signal. Spectrum D is of the clean green PPG signal. It is obtained at the output of the C-ANC after all noise frequency components were eliminated.

Motivated by the architecture in [29], we employ a Cascading Adaptive Noise Cancellation (C-ANC) architecture to remove all the elements of the set $N_{noise}=\left\{f_{n_{i}}|1\leq i\leq N_{n}\right\}$ (developed in Section 2.3.3) from the green PPG signal, *one element at the time*. The block diagram of the proposed C-ANC is shown in Figure 9. We show the frequency spectrum of the input signal in Figure 9 (spectrum A). This is the green signal collected from participant 3 around time 66 s. The spectrum contains three noise frequency components that we wish to eliminate from the signal. The signal collected at the output of the C-ANC (spectrum D in Figure 9) does not contain any of the noise components; only the HR frequency component remained in the spectrum.

A total of $N_{n}$ C-ANC were used to remove the noise components of $N_{noise}$ from the green PPG signal. At the $i^{th}$ stage ($1\leq i\leq N_{n}$), the noise reference signal is a pure sinusoid of frequency $f_{n_{i}}$. For instance, the first ANC filter block shown in Figure 9 removes the first noise frequency component $f_{n_{1}}$ from the normalized green PPG signal (see spectrum B of Figure 9). The output of the first block is denoted $G_{PPG,\;1}$. $G_{PPG,\;1}$ is fed to the next block where the second noise frequency component $f_{n_{2}}$ is removed (see spectrum C of Figure 9). The process is repeated until all noise components are removed from the normalized green PPG signal. The final output, $G_{PPG,\;N_{n}}$, is a noise-free version of the green PPG signal. In the proposed method, the QR-decomposition-based least-squares lattice (QRD-LSL) adaptive filter algorithm was used to remove noise components from the green PPG signal [30]. The method incorporates the desirable features of recursive least-square estimation (fast convergence rate), QR-decomposition (numerical stability), and lattice structure (computational efficiency) [12]. The implementation of the QRD-LSL filter in our study used the built-in MATLAB function “*AdaptiveLatticeFilter*” [31] with 10 filter taps and forgetting factor of 0.99.

#### 2.3.5. Heart Rate Estimation

In this stage (see block E of Figure 3), the green PPG signal is used to compute an HR value. If no noise was detected in the green PPG ($D_{0}=0$), then the normalized green PPG is used for heart rate calculation. When noise was detected in the green PPG signal, a HR value is obtained from the denoised green signal (obtained at the output of block D in Figure 3, also shown in Figure 9). The “Heart Rate Estimation” stage comprises two steps, namely, “Initialization” and “Heart Rate Calculation”.

Initialization (block E1 of Figure 3). This is a process of capturing a baseline HR at rest. In our experiment, it was a one-minute phase during which participants were asked to remain steady in order to capture noise-free green and IR PPG signals. To calculate the initial HR estimate, $\hat{HR}\left(1\right)$ at time step l=1, we used the frequency spectrum of the normalized green PPG signal. $\hat{HR}\left(1\right)$ corresponds to the highest peak within the initial search range 0.5 to 3 Hz (which corresponds to 30 to 180 BPM).

Heart Rate Calculation (block E2 of Figure 3). At time step l+1, the heart rate calculation method we propose employs the following variables in order to generate an HR estimate, $\hat{HR}\left(l+1\right)$:

1. The heart rate estimated from the previous time step *l*, $\hat{HR}\left(l\right)$.
2. A heart rate candidate $HR_{cand}\left(l+1\right)$ which is obtained from the spectrum of the green PPG signal.
3. A heart rate prediction, ${HR}_{pred}\left(l+1\right)$ which is obtained from the long-term (LT) trend of the past six (6) HR estimates. The LT trend is obtained using STL, the Seasonal-Trend decomposition using LOESS (locally estimated scatterplot smoothing) [32]. In this study, we used the MATLAB implementation, *trenddecomp*.

First, we seek to find an HR candidate, $HR_{cand}\left(l+1\right)$, within the wide search range $\Delta_{w}\left(l+1\right)$, which corresponds to the highest peak in the green spectrum ($HR_{cand}\left(l+1\right)\in \left[{\hat{HR}}^{\left(6\right)}\left(l\right)\pm \Delta_{w}\left(l+1\right)/2\right]$). If $HR_{cand}\left(l+1\right)$ is available, we calculate $\delta_{e}\left(l+1\right)$, which is the absolute difference between $HR_{cand}\left(l+1\right)$ and $\hat{HR}\left(l\right)$ (in Hz) at time step l+1. We distinguish between four (4) cases.

**Case** **1.** *If a peak is found in* $\left[{\hat{HR}}^{\left(6\right)}\left(l\right)\pm \Delta_{w}\left(l+1\right)/2\right]$ *and* $D_{0}=0$ *(“no noise”) OR If a peak is found in* $\left[{\hat{HR}}^{\left(6\right)}\left(l\right)\pm \Delta_{w}\left(l+1\right)/2\right]$ *and* $D_{0}=1$ *(“noise is present”) and* $\delta_{e}\left(l+1\right)<0.1$ *Hz.*

*In this case,*$HR_{cand}\left(l+1\right)$*, corresponds to the highest peak in the green spectrum, within the wide search range*$\Delta_{w}\left(l+1\right)$ ($HR_{cand}\left(l+1\right)\in \left[{\hat{HR}}^{\left(6\right)}\left(l\right)\pm \Delta_{w}\left(l+1\right)/2\right]$*). The estimated heart rate,* $\hat{HR}\left(l+1\right)$ *is calculated as*

(5) $$\hat{HR}\left(l+1\right)=HR_{cand}\left(l+1\right).$$

**Case** **2.** *If a peak is found in* $\left[{\hat{HR}}^{\left(6\right)}\left(l\right)\pm \Delta_{w}\left(l+1\right)/2\right]$ *and* $D_{0}=1$ *and* $\delta_{e}\left(l+1\right)>0.1$ *Hz.*

*In this case, we follow the procedure recommended in [7] to consider at most three dominant peaks in the green spectrum, whose magnitude exceed 50% of the maximum peak. Here,* $HR_{cand}\left(l+1\right)$ *is obtained by averaging all the peaks that we considered. The estimated heart rate,* $\hat{HR}\left(l+1\right)$ *is calculated as*

(6) $$\hat{HR}\left(l+1\right)=\beta \times {HR}_{cand}\left(l+1\right)+\left(1-\beta \right)\times HR_{pred}\left(l+1\right),$$

*where* β *is a constant we set to 0.9.*

**Case** **3.** *If no peak is found in*$\left[{\hat{HR}}^{\left(6\right)}\left(l\right)\pm \Delta_{w}\left(l+1\right)/2\right]$.

*In this case, we extend the wide search range,*$\Delta_{w}\left(l+1\right)$*. The extended wide search range is*$\Delta_{w}^{+}\left(l+1\right)=\left(1+\lambda \right)\times \Delta_{w}\left(l+1\right)$ (λ=0.25 *in this study). We seek to find at most three dominant peaks within the extended wide search range,* $\Delta_{w}^{+}\left(l+1\right)$ *(the range* $\left[{\hat{HR}}^{\left(6\right)}\left(l\right)\pm \Delta_{w}^{+}\left(l+1\right)/2\right]$*). If we find at least one peak, we consider at most three dominant peaks, whose magnitude exceed 50% of the maximum peak.* $HR_{cand}\left(l+1\right)$ *is obtained by averaging all the peaks that we considered. The estimated heart rate,* $\hat{HR}\left(l+1\right)$ *is calculated as*

(7) $$\hat{HR}\left(l+1\right)=\beta \times {HR}_{cand}\left(l+1\right)+\left(1-\beta \right)\times HR_{pred}\left(l+1\right),$$

*where* β *is a constant we set to 0.9.*

**Case** **4.** *If no peak is found in*$\left[{\hat{HR}}^{\left(6\right)}\left(l\right)\pm \Delta_{w}\left(l+1\right)/2\right]$*or in*$\left[{\hat{HR}}^{\left(6\right)}\left(l\right)\pm \Delta_{w}^{+}\left(l+1\right)/2\right]$.

*In this case,* $\hat{HR}\left(l+1\right)$ *is calculated as*

(8) $$\hat{HR}\left(l+1\right)=HR_{pred}\left(l+1\right).$$

The heart rate calculation process we used requires the availability of the previous six HR estimates in order to generate an HR prediction, $HR_{pred}\left(l+1\right)$ at time step l+1. Therefore, from time steps l=2 to l=6, the HR estimates $\hat{HR}\left(2\right)$ through $\hat{HR}\left(6\right)$ corresponds to the highest peak in the green spectrum, within the wide search range $\Delta_{w}\left(l+1\right)$ ($\hat{HR}\left(l+1\right)\in \left[{\hat{HR}}^{\left(6\right)}\left(l\right)\pm \Delta_{w}\left(l+1\right)/2\right]$). If no such peak is detected, we increment $\Delta_{w}\left(l+1\right)$ by 0.02 Hz (or 1.2 BPM) and we search again for a peak. This process repeats until a peak is found. ${\hat{HR}}^{\left(6\right)}\left(l\right)$ is the average of all the previously calculated HR estimates (see Equation (3)).

### 2.4. Alternative HR Calculation Methods

In most studies involving PPG signals collected from humans in motion, suitable reference signals, representing motion artifacts, were obtained through additional hardware [28]. For example, when the PPG sensor is mounted on the wrist of a running participant, accelerometer sensors mounted on the participant’s wrist are often used as noise reference signals [33,34,35].

TROIKA is an HR calculation framework proposed by Zhang et al. [7]. TROIKA is based on Singular Spectrum Analysis (SSA) [36] followed by Sparse Signal Reconstruction (SSR) [37] to eliminate the noise dominant components present in PPG signals. The inputs to TROIKA are a green PPG signal and X, Y, and Z accelerometer data. The output is an HR estimate. In our implementation of TROIKA, the noise components were obtained from a tri-axial accelerometer. In [7], TROIKA was tested on data collected from a wrist-worn sensor (that encloses a green PPG channel and X, Y, and Z accelerometer data) from twelve (12) participants, during fast running at peak speed of 15 km/h. The heart rate average absolute error of TROIKA in this test was 2.34 beat per minutes (BPM).

A related method is based on Zhang’s Joint Sparse Spectrum Reconstruction (JOSS). It was shown in [8] to exhibit a heart rate average absolute error as small as 1.28 BPM when tested on the same twelve (12) participants used in Zhang’s TROIKA study [7]. In JOSS, the input signals are a green PPG signal and X, Y, and Z accelerometer data. The accelerometer data are considered the noise signals. The output is an HR estimate. Compared to TROIKA where PPG and accelerometer signals were sampled at 125 Hz, JOSS’s low-sampling rate, namely 25 Hz, is an attractive feature that gives JOSS the potential to be implemented in Very Large-Scale Integration (VLSI) or Field Programmable Gate Array (FPGA) in wearable devices [8].

The HR calculation mechanism of the DWL method was inspired by that of TROIKA and JOSS. We compare the quality of HR calculated by the DWL method which does not require accelerometers, to our implementation of the accelerometer-dependent TROIKA and JOSS. The TROIKA and JOSS experimental results were obtained from the same participants that we employed in the analysis of the DWL method.

## 3. Results

In this section, the results of the HR values calculated using the DWL framework of Section 2.3 are computed and analyzed. First, we define (in Section 3.1) the performance metrics used to compare the performance of the DWL method to that of our implementation of TROIKA and JOSS. In Section 3.2, we assess the performance of the DWL method (using the performance metrics of Section 3.1) on data collected from the participants’ wrists. This comparison is made with respect to (1) the HR ground truth computed from an ECG signal, and (2) the HR levels obtained using TROIKA and JOSS. Lastly, in Section 3.3, we validate the DWL framework by comparing its performance on experimental data collected from the palms (instead of the wrists) of the same participants during a second run (validation run).

### 3.1. Performance Metrics

To assess, evaluate, and compare the HR estimation performance of DWL method to TROIKA and JOSS, we used four metrics, namely; Mean Absolute Error (MAE) (Equation (9)); Mean Absolute Error Percentage (MAEP) (Equation (10)); a specific performance index (PI) [38] (Equation (11)) which is the frequency, in percent, of obtaining an HR estimate that is within ±5 BPM of the HR ground truth; and computation time (CT). We defined CT to be the total time duration (in seconds) that an algorithm takes to generate heart rate levels from the entire 360-second-long off-line data that has already been collected during the experimental run. We compare the HR values calculated by the three tested methods to ground truth values obtained from an ECG signal that is simultaneously recorded, hence synced, with the green and IR PPG waveforms and the X, Y, and Z accelerometer data. All *R* peaks in the ECG signal were manually labeled. The ground truth HR was obtained using Equation (1). The relevant definitions are:

(9) $$MAE\;\left(in\;BPM\right)=\frac{1}{L}\sum_{l=1}^{L}\Delta \left(l\right)\;\;\;\;\;\;\;\;\;\;\;\;\;\;\;\;\;\;\;\;\;\;\;\;\;(\mathrm{ideally}\;0\;\mathrm{BPM})$$

(10) $$MAEP\;\left(in\;\%\right)=\frac{1}{L}\sum_{l=1}^{L}\frac{\Delta (l)}{BPM_{GT}\left(l\right)}\times 100\;\;\;\;\;\;\;\;\;\;\;\;(\mathrm{ideally}\;0\%)$$

(11) $$PI\;\left(in\;\%\right)=\sum_{l=1}^{L}\frac{1_{\left(\Delta \left(l\right)<\epsilon_{1}\right)}}{L}\times 100\;\;\;\;\;\;\;\;\;\;\;\;\;\;\;\;\;\;\;\;\;\;\;\;(\mathrm{ideally}\;100\%)$$

In Equations (9)–(11), Δ(l) is defined as

(12) $$\Delta \left(l\right)\;\left(in\;BPM\right)=|BPM_{HR\;method}\left(l\right)-BPM_{GT}\left(l\right)|\;,$$

where |.| is the absolute value. $BPM_{HR\;method}\left(l\right)$ is the HR in beat per minutes (BPM) calculated using each one of the tested methods (DWL, TROIKA, and JOSS) at time step *l*. $BPM_{GT}\left(l\right)$ is the HR ground truth value in BPM obtained as $BPM_{GT}\left(l\right)=HR_{GT}\left(l\right)\times 60$, where $HR_{GT}\left(l\right)$ is calculated using Equation (1). In Equation (11), 1 is the indicator function that returns 1 if $\Delta \left(l\right)<\epsilon_{1}$ and 0 otherwise. $\epsilon_{1}$ was set to 5 BPM. HR estimated using DWL at time step *l* (in BPM) is calculated as $BPM_{DWL}\left(l\right)={\hat{HR}}^{\left(3\right)}\left(l\right)\times 60$, where ${\hat{HR}}^{\left(3\right)}\left(l\right)$ is the 3-point-averaged HR estimate (see Equation (3)).

### 3.2. DWL Performance on Wrist Data

Data were collected from fourteen (14) participants while standing, walking, and running on the treadmill, following the experimental protocol described in Section 2.1. In this section, we analyze data collected from participants 1 to 11. Data from participants 12, 13, and 14 are not included in our analysis since for these participants, the system suffered from physical malfunction (intermittent readings due to loss of sensor contact). However, we still provide the data for these participants in the repository in [16]. Every 2 s, the preceding 8-second-long green and IR PPG data were used to generate a short-term 3-point-average HR estimate, ${\hat{HR}}^{\left(3\right)}\left(l\right)$, using the DWL method. HR levels obtained using DWL are compared to those of TROIKA and JOSS.

For the TROIKA implementation, we used a sampling rate of 100 Hz. We recreated the TROIKA code using MATLAB. Our code was tested on the same dataset of the TROIKA paper and compared to the results presented in [7]. The results using our code are very close to the results presented in the TROIKA paper.

For the JOSS implementation, we used a sampling rate of 25 Hz, as suggested in the JOSS paper [8]. We recreated the JOSS code using MATLAB. Our code was tested on the dataset used in JOSS paper and compared to the results presented in [8]. The results using our code are very close to the results presented in the JOSS paper.

**Figure 10:**
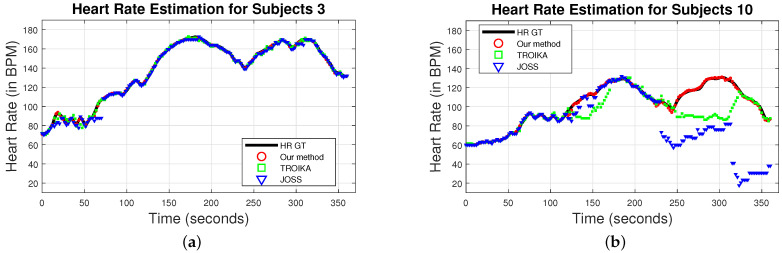
HR calculated for the whole experimental run using DWL method (red circles), TROIKA (green squares), and JOSS (blue triangles). (**a**) HR values for participant 3. DWL, TROIKA, and JOSS were able to calculate accurate heart rate levels. (**b**) HR values for participant 10. TROIKA lost track of the correct heart rate from 120 to 175 s and from 250 to 325 s. Similary, JOSS lost track of the correct heart rate from 225 s until the end of the experimental run. DWL methods was able to estimate the participant’s HR accurately during the whole experimental run.

As examples, we show in Figure 10 the HR calculated for the whole experimental run for two participants, participant 3 (Figure 10a) and participant 10 (Figure 10b). We use red circles, green squares, and blue triangles to represent the HR values calculated using DWL, TROIKA, and JOSS, respectively. The ground truth HR is the solid black line. In Figure 10a all three methods generate accurate HR estimates (the magnitude of the noise level present in the signals of participant 3 was small). For participant 10 (see Figure 10b), however, TROIKA lost track of the correct heart rate from 120 to 175 s and from 250 to 325 s. This phenomenon (losing track of the correct HR) is referred to as Lock Loss. Similary, JOSS suffered from a Lock Loss from 225 s until the end of the experimental run. During these intervals, the DWL method was still able to estimate the participant’s HRs accurately (see red circles of Figure 10b).

We calculate MAE, MAEP, PI, and CT for all eleven (11) experimental participants and present them in Table 2, Table 3, Table 4 and Table 5, respectively. In Table 2, we show the MAE for DWL, TROIKA, and JOSS. We calculate and report the MAE mean and standard deviation for each method in the second to last row of Table 2. In the last row of Table 2, we calculate the MAE mean and standard deviation of all participants that do not suffer from Lock Loss. Lock Loss happens if MAE exceeds 5 BPM. Participants who suffer Lock Loss are underlined. Table 3 summarizes the MAEP for DWL, TROIKA, and JOSS. We calculate and report the MAEP mean and standard deviation for each method in the last row of Table 3. Moreover, we calculate PI for DWL, TROIKA, and JOSS, and report it in Table 4. In the last row of Table 4, we calculate the PI mean and standard deviation of all participants.

As shown in Table 2 the average MAE for all eleven participants using the DWL method is MAE of 1.22|0.57 BPM (“mean|standard deviation”) (see Table 2), which is smaller than average MAE of TROIKA (3.24|2.82 BPM) and JOSS (11.98|25.79 BPM), respectively. When we exclude participants who suffer from Lock Loss (shown in the last row of Table 2), DWL (with the same average MAE = 1.22|0.57 BPM) still yields a smaller average MAE than that of TROIKA (with average MAE = 2.05|1.03 BPM) and JOSS (with average MAE = 2.11|1.24 BPM). Note that the MAE calculated using DWL method did not exceed 5 BPM for any of the participants. However, this was not the case for TROIKA and JOSS method. Participant 10 presents an example where the MAE of TROIKA (9.34 BPM) and JOSS (21.8 BPM) exceeds 5 BPM, whereas the MAE of the DWL method is 0.85 BPM.

In addition to MAE, we calculate average MAEP of all three methods for all eleven participants. Average MAEP of DWL method of 0.95|0.38% is smaller than average MAEP of TROIKA (2.58|2.19%) and JOSS (8.68|17.6%) (see Table 3).

Table 4 summarizes the PI values for all eleven (11) participants. The PI of DWL method is larger than the PI of TROIKA and JOSS. For instance, on average, the PI of DWL method is 95.88|4.9% that is greater than that of TROIKA with 83.87|12.75% and JOSS with 78.62|26.16%.

The CT is an indication of the algorithm’s computational complexity. In order to be implement in wearable devices, the algorithm should be able to run in real-time and be energy efficient. A desirable algorithm should have a small CT. Table 5 shows the CT of DWL, TROIKA, and JOSS for participants 1 to 11. The average CT of DWL is smaller than that of TROIKA and JOSS. For instance, the average CT of DWL is 3.0|0.3 s is smaller than the average CT of TROIKA with 247.7|43.8 s and JOSS with 8.5|0.24 s.

**Figure 11:**
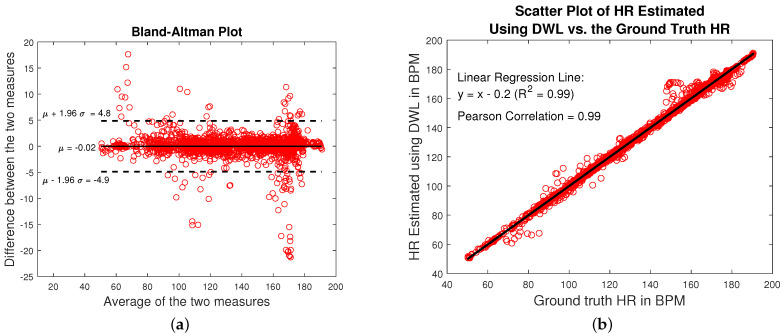
(**a**) Bland–Altman plot of HR estimated using DWL method and the ground truth HR for participants one (1) to eleven (11). The LOA = [−4.9, 4.8] BPM. (**b**) Scatter plot of HR estimated using DWL method (on the y-axis) vs. the ground truth HR (x-axis) for participants one (1) to eleven (11). The linear regression line that fits the data is shown in black. The line is y = x − 0.2 ($R^{2}=0.99$). The Pearson correlation is found to be 0.99.

Additionally, we show the *Bland–Altman plot* (Figure 11a) of the HR values computed using DWL method for participants one (1) through eleven (11). The Bland–Altman plot describes the agreement between two quantitative measurements (A and B) by constructing the Limits of Agreements (LOA). These statistical limits are calculated by using the mean and the standard deviation of the differences between the two measurements. The resulting graph is a scatter plot, in which the y-axis shows the difference between the two paired measurements (A − B) and the x-axis represents the average of these measures ((A + B)/2) [39]. The LOA we use is [μ−1.96×σ, μ+1.96×σ] (1.96×σ corresponds to 95% confidence level) where μ is the average difference between each HR estimate and the associated ground-truth HR against their average, and σ is the standard deviation [7]. The LOA in Figure 11a is [−4.9, 4.8] BPM. Moreover, we construct the scatter plot of the HR estimated using DWL method versus the associated ground truth HR for participants one (1) through eleven (11). The scatter plot is shown in Figure 11b. We construct a linear regression for the data points of Figure 11b. The fitted line is y = x − 0.2 ($R^{2}=0.99$), where x is the ground truth HR and y is the HR estimated using DWL method. The Pearson correlation between the HR estimated using DWL method and ground truth HR is also calculated and found to be 0.99. The high $R^{2}$ value and Pearson correlation indicate that DWL method is able to compute accurate HR levels.

### 3.3. Validation of the DWL Method on Palm Data

In order to validate the performance of the DWL framework, we ran a second experiment (validation run). During the second experiment, we asked the same volunteers who participated in our previous experiment to run on the treadmill again, following the experimental protocol described in Section 2.1. We reused the same ECG, accelerometer, and PPG sensors. The only difference was that we mount the dual wavelength sensor onto the participant’s palm (instead of wrist). Data for all participants are provided in the repository in [16].

Both wrist and palm experiments took place on the same day. There was a break of approximately 15 min between the first and the second run during which the dual-wavelength PPG sensor was relocated from the wrist to the palm of the participant. Participants 5 and 13 deviated from the data collection protocol by interfering with the sensor during collection. Their measurements were excluded from the analysis we provide (but are available in the repository in [16]).

MAE, MAEP, and PI were calculated from the twelve (12) participants of the “palm run” for DWL, TROIKA, and JOSS. We show in Table 6, the summary of the performance metrics (MAE, MAEP, and PI) obtained for the first run (the “wrist run”), and the second run (the “palm run”). The results are presented as “mean|standard deviation”.

Table 6 shows that the DWL method performs as well when the measurements were taken from the wrist as when they were taken from the palm.

## 4. Discussion

We presented a framework for heart rate (HR) calculation under motion using a dual-wavelength (green and IR) PPG sensor. We used PPG data collected from 14 individuals engaged in high-intensity full-body exercise. Analysis of green and IR PPG signals indicates that the IR PPG signal is a good noise reference signal. We employed this observation to develop a motion-resistant HR calculation method derived from [9] that measures noise components from the IR PPG signal. Afterwards, a green PPG signal is denoised and used for HR calculation. The proposed method, Dual Wavelength (DWL), was tested on experimental data collected from participants’ wrists while the participants were standing, walking, and running on a treadmill. The performance of the method, using several measures of accuracy and computational effort, was then compared to popular methods in the literature that use data from a tri-axial accelerometer for denoising, namely TROIKA and JOSS. Using the experimental wrist-data we collected, we showed that the DWL method exhibits good performance in the face of motion artifacts. For instance, DWL yielded a Mean Absolute Error (MAE) of 1.22|0.57 BPM, Mean Absolute Error Percentage (MAEP) of 0.95|0.38%, and performance index (PI) (which is the frequency in percent of the event that we obtain an HR estimate that is within ±5 BPM of the HR ground truth) of 95.88|4.9%. Moreover, DWL yielded a short computation period of 3.0|0.3 s to process a 360-second-long run. We validated the performance of the DWL method by testing it on data collected from the participants’ palms, obtaining similar behavior. The DWL method is desirable since (1) it performed well under high-intensity full-body repetitive “macro-motion”, exhibiting high accuracy in the presence of motion artifacts (as compared to the leading accelerometer-dependent HR calculation techniques TROIKA and JOSS); (2) it used only PPG signals; auxiliary signals such as accelerometer signals were not needed; and (3) it was computationally efficient, hence implementable in wearable devices.

## Figures and Tables

**Table 1:** Instruments used for data collection in the exercise experiment.

Instrument/Sensor	Manufacturer	Reference
Split-belt Instrumented Treadmill	Bertec Corp. (Columbus, OH, USA)	Catalog in [14]
IR LED (TSAL6100)	Vishay Intertechnology Inc. (Malvern, PA, USA)	Datasheet in [17]
Green LED (A-U5MUGC12)	Light House LEDs LLC (Medical Lake, WA, USA)	Datasheet in [18]
Photo-detector (OPT101)	Texas-Instrument Inc. (Dallas, TX, USA)	Datasheet in [19]
Delsys Trigno Avanti (tri-axial accelerometer)	Delsys Inc. (Natick, MA, USA)	Catalog in [20]
Trigno EKG Biofeedback sensor (ECG)	Delsys Inc.	Catalog in [21]

**Table 2:** MAE in BPM for all eleven (11) experimental participants, using DWL, TROIKA, and JOSS (ideal MAE is 0). The second to last row shows the MAE average of all eleven (11) participants shown as “mean|standard deviation”. The last row shows the MAE average of participants that do not suffer from Lock Loss (MAE value is less than 5 BPM). We underline the MAE values that exceed 5 BPM.

	HR Calculation Methods		
**Participant Number**	**TROIKA** ${\;}^{1}$	**JOSS** ${\;}^{1}$	**DWL** ${\;}^{2}$
1	1.09	1.39	0.74
2	4.3	87.61	1.48
3	1.21	1.41	0.63
4	1.96	1.72	2.36
5	7.85	5.49	1.86
6	2.57	2.73	1.18
7	1.83	2.03	1.64
8	1.08	0.84	0.61
9	1.73	1.86	0.76
10	9.34	21.8	0.85
11	2.72	4.87	1.31
Average	3.24 | 2.82 BPM	11.98 | 25.79 BPM	1.22 | 0.57 BPM
Average without Lock Loss ${}^{*}$	2.05 | 1.03 BPM	2.11 | 1.24 BPM	1.22 | 0.57 BPM

${}^{1}$ JOSS and TROIKA use tri-axial accelerometer data as noise reference. ${}^{2}$ DWL uses an IR PPG signal as noise reference. ${}^{*}$ All MAE values that exceed 5 BPM are underlined and not included into the calculation of the average performance.

**Table 3:** MAEP in % for all eleven (11) experimental participants, using DWL, TROIKA, and JOSS (ideal MAEP is 0%). The last row shows the MAEP average of all eleven (11) participants shown as “mean|standard deviation”.

	HR Calculation Methods		
**Participant Number**	**TROIKA**	**JOSS**	**DWL**
1	0.92	1.19	0.66
2	3.88	59.22	1.47
3	1.03	1.23	0.5
4	1.43	1.31	1.61
5	4.96	3.41	1.19
6	2.06	2.24	0.85
7	1.38	1.49	1.16
8	0.77	0.62	0.42
9	1.94	2.08	0.81
10	7.91	19.53	0.83
11	2.07	3.19	1.00
Average	2.58 | 2.19%	8.68 | 17.6%	0.95 | 0.38%

**Table 4:** PI in % for all eleven (11) experimental participants, using DWL, TROIKA, and JOSS (ideal PI is 100%). The last row shows the PI average of all eleven (11) participants shown as “mean|standard deviation”.

	HR Calculation Methods		
**Participant Number**	**TROIKA**	**JOSS**	**DWL Method**
1	96.02	93.75	98.36
2	72.57	6.86	94.29
3	96.02	93.75	100
4	88.07	90.91	86.93
5	61.58	80.79	88.7
6	80.68	84.66	100
7	90.96	88.7	91.53
8	97.18	98.31	100
9	90.91	89.2	100
10	63.84	57.63	99.44
11	84.75	80.23	95.48
Average	83.87 | 12.75%	78.62 | 26.16%	95.88 | 4.9%

**Table 5:** CT in seconds for all eleven (11) experimental participants, using DWL, TROIKA, and JOSS. The last row shows the CT average of all eleven (11) participants shown as “mean|standard deviation”.

	HR Calculation Methods		
**Participant Number**	**TROIKA**	**JOSS**	**DWL Method**
1	243.6	8.5	2.8
2	238.0	8.3	3.0
3	294.6	8.6	3.1
4	259.7	8.4	2.7
5	246.5	8.5	3.1
6	239.7	9.2	3.7
7	237.0	8.5	2.7
8	326.4	8.5	2.8
9	278.3	8.3	2.8
10	194.3	8.5	3.3
11	166.9	8.5	3.1
Average	247.7 | 43.8 s	8.5 | 0.24 s	3.0 | 0.3 s

The results were generated by MATLAB R2022b on a personal computer, with an Intel^®^Core*^TM^* i9-10900K CPU running at 3.70 GHz, 32GB RAM, and Windows 11 operating system.

**Table 6:** Summary of performance metrics for run 1 (wrist run) and run 2 (validation palm run). For run 1, we showed the average performance of eleven (11) participants. For run 2, we showed the average performance of twelve (12) participants. Results are represented as “mean|standard deviation”.

	Run 1 (Wrist Run)			Run 2 (Palm Run)		
	**TROIKA**	**JOSS**	**DWL**	**TROIKA**	**JOSS**	**DWL**
Average MAE (BPM) of all participants	3.24|2.82	11.98|25.79	1.22|0.57	1.79|0.92	12.88|27.41	1.3|0.77
Average MAE (BPM) of participants without Lock Loss	2.05|1.03	2.11|1.24	1.22|0.57	1.79|0.92	1.57|0.83	1.3|0.77
Average MAEP (%) of all participants	2.58|2.19	8.68|17.6	0.95|0.38	1.43|0.69	8.51|17.62	1.01|0.6
Average PI (%) of all participants	83.87|12.75	78.62|26.16	95.88|4.9	90.23|8.94	80.93|29.18	95.33|6.46

## Data Availability

The raw sensors data used in this study—namely, ECG signal, X, Y, and Z accelerometer readings, and green and infrared PPG signals—are openly available in the Github repository at https://github.com/ludvikalkhoury/DWL-Method.git (accessed on 1 December 2022) [16]. We also provide gyroscope tri-axial data that were recorded from the Delsys Trigno Avanti sensor for all fourteen participants during the wrist and palm runs. The gyroscope data were not used in this work. Moreover, the Github repository provides the MAE, MAEP, and PI metrics of the DWL method from all fourteen participants during the wrist and palm runs. These metrics were also calculated using our implementations of TROIKA and JOSS.

## Appendix A. Wide, Medium, and Narrow Search Ranges

In Section 2.3, we introduced three search zones, namely; the “narrow search range”, $\Delta_{n}\left(l+1\right)$; the “medium search rang”, $\Delta_{m}\left(l+1\right)$; and the “wide search range”, $\Delta_{w}\left(l+1\right)$. $\Delta_{m}\left(l+1\right)$ and $\Delta_{n}\left(l+1\right)$ are around $\hat{HR}\left(l\right)$, and $\Delta_{w}\left(l+1\right)$ is around ${\hat{HR}}^{\left(6\right)}\left(l\right)$. ${\hat{HR}}^{\left(6\right)}\left(l\right)$ is the average of the 6 previous heart rate (HR) estimates which is obtained using Equation (3).

Wide search ranges, $\Delta_{w}\left(l+1\right)$: $\Delta_{w}\left(l+1\right)$ is adopted from [29] and employed in the HR estimation process (Section 2.3.5) as the range in which we search for the participant’s HR. At time step l+1, the search for $\hat{HR}\left(l+1\right)$ is confined to

(A1) $$[{\hat{HR}}^{\left(6\right)}\left(l\right)-\frac{\Delta_{w}\left(l+1\right)}{2},\;{\hat{HR}}^{\left(6\right)}\left(l\right)+\frac{\Delta_{w}\left(l+1\right)}{2}]\;Hz\;,$$

$\Delta_{w}\left(l\right)$ is defined as

(A2) $$\Delta_{w}\left(l\right)=\left\{\begin{array}{cc} c_{1}+2\times max\left(\hat{HR}\left(j\right)-\hat{HR}\left(j-1\right)\;|\;l-k\leq j\leq l-1\right) & ,\mathrm{if}\;l>k\\ c_{0} & ,\mathrm{if}\;l\leq k\;, \end{array}\right.$$

where max(.) is the maximum value. $\Delta_{w}\left(l\right)$ is updated based on the maximum value of the differences between two consecutive HR values calculated over *k* previous time steps. For the first *k* iterations, $\Delta_{m}\left(l\right)$ is equal to $c_{0}$. In this work, $c_{0}$ was set to 0.33 Hz (or 20 BPM), $c_{1}$ was set to 0.37 Hz (or 22 BPM), and k=15 (which is equivalent to 30 s).

Medium and narrow search range, $\Delta_{m}\left(l+1\right)$ and $\Delta_{n}\left(l+1\right)$: $\Delta_{m}\left(l+1\right)$ and $\Delta_{n}\left(l+1\right)$ are employed in the motion-artifact frequency components identification process (Section 2.3.3). At time step l+1, the *medium search range* is the range confined to

(A3) $$[\hat{HR}\left(l\right)-\frac{\Delta_{m}\left(l+1\right)}{2},\;\hat{HR}\left(l\right)+\frac{\Delta_{m}\left(l+1\right)}{2}]\;Hz\;,$$

where $\hat{HR}\left(l\right)$ is the HR estimated at the time step *l*. $\Delta_{m}\left(l\right)$ is defined as

(A4) $$\Delta_{m}\left(l\right)=\left\{\begin{array}{cc} c_{1}+2\times SD\left(\hat{HR}\left(j\right)-\hat{HR}\left(j-1\right)\;|\;l-k\leq j\leq l-1\right) & ,\mathrm{if}\;l>k\\ c_{0} & ,\mathrm{if}\;l\leq k \end{array}\right.\;\;,$$

where SD(.) is the standard deviation. $\Delta_{m}\left(l\right)$ is updated based on the standard deviation of the differences between two consecutive HR values calculated over *k* previous time steps. For the first *k* iterations, $\Delta_{w}\left(l\right)$ is equal to constant $c_{0}$. In this work, the parameters $c_{0}$, $c_{1}$, and *k* are same defined for the *wide search range*, namely; $c_{0}=0.33$ Hz (or 20 BPM), $c_{1}=0.37$ Hz (or 22 BPM), and k=15 (which is equivalent to 30 s).

The *narrow search range* is defined as $\Delta_{n}\left(l+1\right)=\frac{\Delta_{m}\left(l+1\right)}{2}$.
